# 4-(3,5-Dioxo-10-oxa-4-aza­tricyclo­[5.2.1.0^2,6^]decan-4-yl)-10-oxa-4-aza­tricyclo­[5.2.1.0^2,6^]decane-3,5-dione

**DOI:** 10.1107/S1600536812000542

**Published:** 2012-01-14

**Authors:** Peng-Peng Wang, Qiu-Yue Lin, Fan Zhang

**Affiliations:** a Zhejiang Key Laboratory for Reactive Chemistry on Solid Surfaces, Institute of Physical Chemistry, Zhejiang Normal University, Jinhua, Zhejiang 321004, People’s Republic of China; b College of Chemistry and Life Science, Zhejiang Normal University, Jinhua 321004, Zhejiang, People’s Republic of China

## Abstract

In the title compound, C_16_H_16_N_2_O_6_, the dihedral angle between the two pyrrolidine rings is 79.38 (14)°.

## Related literature

Norcantharidin [systematic name: 7-oxabicyclo­(2.2.1)heptane-2,3-dicarb­oxy­lic anhydride] and its derivatives are of significant inter­est as serine/threonine protein phosphatase 1 and 2A inhibitors, see: Hill *et al.* (2008 ▶). For related structures, see: Li *et al.* (2011 ▶); Zhu & Lin (2009 ▶).
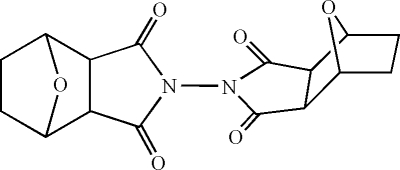


## Experimental

### 

#### Crystal data

C_16_H_16_N_2_O_6_
*M* *_r_* = 332.31
Orthorhombic, 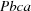

*a* = 10.2342 (6) Å
*b* = 10.5673 (6) Å
*c* = 27.3485 (17) Å
*V* = 2957.7 (3) Å^3^
*Z* = 8
Mo *K*α radiation
μ = 0.12 mm^−1^
*T* = 296 K
0.14 × 0.09 × 0.08 mm

#### Data collection

Bruker P4 diffractometer
Absorption correction: multi-scan (*SADABS*; Sheldrick, 1996 ▶) *T*_min_ = 0.987, *T*_max_ = 0.991
43071 measured reflections
3423 independent reflections
1581 reflections with *I* > 2σ(*I*)
*R*_int_ = 0.163

#### Refinement

*R*[*F* ^2^ > 2σ(*F* ^2^)] = 0.083
*wR*(*F* ^2^) = 0.224
*S* = 1.07
3423 reflections
217 parameters
H-atom parameters constrained
Δρ_max_ = 0.24 e Å^−3^
Δρ_min_ = −0.21 e Å^−3^

### 

Data collection: *SMART* (Bruker, 2004 ▶); cell refinement: *SAINT* (Bruker, 2004 ▶); data reduction: *SAINT*; program(s) used to solve structure: *SHELXTL* (Sheldrick, 2008 ▶); program(s) used to refine structure: *SHELXTL*; molecular graphics: *SHELXTL*; software used to prepare material for publication: *SHELXTL*.

## Supplementary Material

Crystal structure: contains datablock(s) I, global. DOI: 10.1107/S1600536812000542/ff2050sup1.cif

Structure factors: contains datablock(s) I. DOI: 10.1107/S1600536812000542/ff2050Isup2.hkl

Supplementary material file. DOI: 10.1107/S1600536812000542/ff2050Isup3.cml

Additional supplementary materials:  crystallographic information; 3D view; checkCIF report

## Figures and Tables

**Fig. 1.**
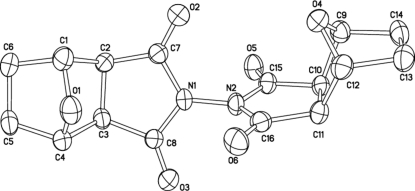
A view of the molecule of the title compound showing the atom-labelling scheme with displacement ellipsoids drawn at 30% probability. Hydrogen atoms were omitted for clarity.

## supplementary crystallographic information

### Comment

Norcantharidin and its derivatives are of significant interest as serine/threonine protein phosphatase 1 and 2A inhibitors (Hill *et al.*, 2008); norcantharidin has been used in the treatment of primary hepatoma and upper gastrointestinal carcinomas, and it does not display the nephrotoxicity of cantharidin. Related norcantharidin imides were reported by Zhu & Lin (2009) and Li *et al.* (2011).

X-ray crystallography confirmed the molecular structure and the atom connectivity for the title compound, as illustrated in Fig. 1. In the molecule, the dihedral angle between the two pyrrolidine rings is 79.38 (14)°. The pyrrolidine rings are linked *via* N—N bond. The bond angles of C7—N1—N2, C8—N1—N2 and C7—N1—C8 are 121.8 (3),123.4 (3) and 114.6 (3), respectively.

### Experimental

A mixture of 0.5 mmol norcantharidin, 0.5 mmol 2-amino-1,3,4-thiadiazole, 0.5 mmol palladium chloride as a promoter, and 10 mL distilled water was sealed in a 25 mL stainless steel reactor with a Telflon liner and heated at 393 K for 3 d. The reactor was cooled slowly to room temperature over 3 d. The solution was filtered and after 3 weeks, crystals with suitable size for single-crystal X-ray diffraction were obtained.

### Refinement

The H atoms were positioned geometrically and refined using a riding model [aliphatic of tertiary carbon C—H = 0.98 Å, aliphatic of secondary carbon C—H = 0.97 Å, *U*_iso_(H) = 1.2*U*_eq_(C)].

### Crystal data

**Table:**

C_16_H_16_N_2_O_6_	*F*(000) = 1392
*M**_r_* = 332.31	*D*_x_ = 1.493 Mg m^−^^3^
Orthorhombic, *P**b**c**a*	Mo *K*α radiation, λ = 0.71073 Å
Hall symbol: -P 2ac 2ab	Cell parameters from 2007 reflections
*a* = 10.2342 (6) Å	θ = 1.5–27.6°
*b* = 10.5673 (6) Å	µ = 0.12 mm^−^^1^
*c* = 27.3485 (17) Å	*T* = 296 K
*V* = 2957.7 (3) Å^3^	Block, colourless
*Z* = 8	0.14 × 0.09 × 0.08 mm

### Data collection

**Table:**

Bruker P4 diffractometer	3423 independent reflections
Radiation source: fine-focus sealed tube	1581 reflections with *I* > 2σ(*I*)
graphite	*R*_int_ = 0.163
ω scans	θ_max_ = 27.6°, θ_min_ = 1.5°
Absorption correction: multi-scan (*SADABS*; Sheldrick, 1996)	*h* = −13→13
*T*_min_ = 0.987, *T*_max_ = 0.991	*k* = −13→13
43071 measured reflections	*l* = −35→35

### Refinement

**Table:**

Refinement on *F*^2^	Primary atom site location: structure-invariant direct methods
Least-squares matrix: full	Secondary atom site location: difference Fourier map
*R*[*F*^2^ > 2σ(*F*^2^)] = 0.083	Hydrogen site location: inferred from neighbouring sites
*wR*(*F*^2^) = 0.224	H-atom parameters constrained
*S* = 1.07	*w* = 1/[σ^2^(*F*_o_^2^) + (0.0862*P*)^2^ + 1.8201*P*] where *P* = (*F*_o_^2^ + 2*F*_c_^2^)/3
3423 reflections	(Δ/σ)_max_ = 0.002
217 parameters	Δρ_max_ = 0.24 e Å^−^^3^
0 restraints	Δρ_min_ = −0.21 e Å^−^^3^

### Special details

**Table:**

Geometry. All e.s.d.'s (except the e.s.d. in the dihedral angle between two l.s. planes) are estimated using the full covariance matrix. The cell e.s.d.'s are taken into account individually in the estimation of e.s.d.'s in distances, angles and torsion angles; correlations between e.s.d.'s in cell parameters are only used when they are defined by crystal symmetry. An approximate (isotropic) treatment of cell e.s.d.'s is used for estimating e.s.d.'s involving l.s. planes.
Refinement. Refinement of *F*^2^ against ALL reflections. The weighted *R*-factor *wR* and goodness of fit *S* are based on *F*^2^, conventional *R*-factors *R* are based on *F*, with *F* set to zero for negative *F*^2^. The threshold expression of *F*^2^ > σ(*F*^2^) is used only for calculating *R*-factors(gt) *etc*. and is not relevant to the choice of reflections for refinement. *R*-factors based on *F*^2^ are statistically about twice as large as those based on *F*, and *R*- factors based on ALL data will be even larger.

### Fractional atomic coordinates and isotropic or equivalent isotropic displacement parameters (Å^2^)

**Table:**

	*x*	*y*	*z*	*U*_iso_*/*U*_eq_	
O4	0.4418 (3)	−0.1479 (3)	0.03560 (11)	0.0595 (8)	
O1	0.5405 (3)	0.4020 (3)	0.13629 (11)	0.0643 (9)	
N1	0.5287 (3)	0.0942 (3)	0.15182 (13)	0.0517 (9)	
O2	0.3401 (3)	0.1174 (3)	0.10792 (12)	0.0658 (9)	
O3	0.7107 (3)	0.1261 (3)	0.19883 (14)	0.0784 (11)	
O5	0.6972 (3)	0.0800 (3)	0.07064 (12)	0.0701 (10)	
C16	0.5130 (4)	−0.1318 (4)	0.14002 (15)	0.0484 (10)	
C11	0.6499 (4)	−0.1471 (3)	0.06726 (14)	0.0486 (10)	
H11A	0.7384	−0.1821	0.0654	0.058*	
N2	0.5691 (3)	−0.0145 (3)	0.12856 (13)	0.0496 (9)	
C12	0.5594 (4)	−0.2230 (3)	0.10115 (14)	0.0446 (10)	
H12A	0.6021	−0.2979	0.1149	0.054*	
O6	0.4401 (3)	−0.1492 (3)	0.17385 (11)	0.0654 (9)	
C7	0.4108 (4)	0.1543 (4)	0.13968 (16)	0.0498 (10)	
C1	0.5826 (4)	0.3963 (4)	0.18576 (17)	0.0557 (12)	
H1A	0.6776	0.4021	0.1896	0.067*	
C8	0.6043 (4)	0.1584 (4)	0.18598 (16)	0.0502 (11)	
C10	0.4465 (4)	−0.2567 (4)	0.06668 (16)	0.0542 (11)	
H10A	0.3640	−0.2742	0.0836	0.065*	
C15	0.6466 (4)	−0.0144 (4)	0.08651 (16)	0.0510 (11)	
C3	0.5256 (4)	0.2710 (4)	0.20309 (16)	0.0491 (10)	
H3A	0.5105	0.2698	0.2385	0.059*	
C2	0.4016 (5)	0.3919 (4)	0.14670 (18)	0.0622 (13)	
H2A	0.3459	0.3956	0.1176	0.075*	
C14	0.4899 (5)	−0.3623 (4)	0.03254 (17)	0.0622 (12)	
H14A	0.4165	−0.3986	0.0150	0.075*	
H14B	0.5357	−0.4286	0.0501	0.075*	
C9	0.5756 (4)	−0.1560 (4)	0.01894 (16)	0.0545 (11)	
H9A	0.5995	−0.0901	−0.0046	0.065*	
C13	0.5819 (5)	−0.2898 (4)	−0.00196 (16)	0.0623 (13)	
H13A	0.6700	−0.3235	−0.0007	0.075*	
H13B	0.5507	−0.2923	−0.0354	0.075*	
C4	0.3968 (4)	0.2652 (4)	0.17362 (16)	0.0500 (11)	
H4A	0.3199	0.2579	0.1948	0.060*	
C6	0.3774 (5)	0.4990 (4)	0.18326 (18)	0.0640 (13)	
H6A	0.3051	0.4795	0.2049	0.077*	
H6B	0.3602	0.5785	0.1667	0.077*	
C5	0.5080 (5)	0.5027 (4)	0.21108 (18)	0.0626 (13)	
H5A	0.5517	0.5835	0.2071	0.075*	
H5B	0.4961	0.4856	0.2456	0.075*	

### Atomic displacement parameters (Å^2^)

**Table:**

	*U*^11^	*U*^22^	*U*^33^	*U*^12^	*U*^13^	*U*^23^
O4	0.057 (2)	0.0543 (18)	0.0669 (19)	0.0109 (15)	−0.0062 (16)	−0.0045 (15)
O1	0.080 (3)	0.0474 (17)	0.065 (2)	−0.0046 (15)	0.0149 (18)	0.0010 (15)
N1	0.056 (2)	0.0350 (18)	0.064 (2)	0.0030 (16)	−0.0021 (18)	−0.0112 (16)
O2	0.058 (2)	0.0620 (19)	0.077 (2)	0.0033 (16)	−0.0110 (18)	−0.0143 (17)
O3	0.066 (2)	0.061 (2)	0.108 (3)	0.0160 (17)	−0.025 (2)	−0.0178 (19)
O5	0.074 (2)	0.0484 (17)	0.088 (2)	−0.0161 (16)	0.0124 (18)	0.0048 (17)
C16	0.057 (3)	0.041 (2)	0.046 (2)	0.004 (2)	0.002 (2)	0.001 (2)
C11	0.057 (3)	0.036 (2)	0.053 (2)	0.0058 (19)	0.004 (2)	0.0009 (18)
N2	0.056 (2)	0.0322 (17)	0.060 (2)	0.0004 (16)	0.0071 (19)	−0.0058 (16)
C12	0.050 (3)	0.0285 (19)	0.055 (2)	0.0028 (18)	0.000 (2)	0.0018 (17)
O6	0.085 (2)	0.0537 (18)	0.0576 (18)	−0.0048 (17)	0.0187 (18)	−0.0052 (15)
C7	0.047 (3)	0.042 (2)	0.061 (3)	−0.003 (2)	0.001 (2)	0.001 (2)
C1	0.051 (3)	0.039 (2)	0.078 (3)	−0.0008 (19)	−0.002 (2)	−0.003 (2)
C8	0.046 (3)	0.039 (2)	0.065 (3)	0.001 (2)	−0.005 (2)	0.000 (2)
C10	0.056 (3)	0.045 (2)	0.062 (3)	−0.005 (2)	0.005 (2)	−0.003 (2)
C15	0.047 (3)	0.043 (2)	0.063 (3)	−0.001 (2)	−0.002 (2)	0.003 (2)
C3	0.057 (3)	0.037 (2)	0.054 (2)	−0.0003 (19)	−0.001 (2)	−0.0046 (19)
C2	0.070 (3)	0.046 (2)	0.070 (3)	0.006 (2)	−0.010 (3)	0.002 (2)
C14	0.079 (3)	0.042 (2)	0.065 (3)	0.000 (2)	−0.003 (3)	−0.012 (2)
C9	0.062 (3)	0.048 (2)	0.053 (2)	0.007 (2)	0.002 (2)	0.005 (2)
C13	0.075 (3)	0.060 (3)	0.052 (3)	0.013 (2)	0.000 (2)	−0.009 (2)
C4	0.042 (2)	0.043 (2)	0.065 (3)	0.0022 (19)	0.001 (2)	−0.002 (2)
C6	0.064 (3)	0.041 (2)	0.087 (3)	0.010 (2)	−0.005 (3)	−0.004 (2)
C5	0.075 (3)	0.038 (2)	0.075 (3)	0.001 (2)	0.001 (3)	−0.011 (2)

### Geometric parameters (Å, °)

**Table:**

O4—C10	1.431 (5)	C1—H1A	0.9800
O4—C9	1.446 (5)	C8—C3	1.511 (6)
O1—C1	1.421 (5)	C10—C14	1.520 (6)
O1—C2	1.454 (6)	C10—H10A	0.9800
N1—N2	1.376 (4)	C3—C4	1.547 (6)
N1—C8	1.390 (5)	C3—H3A	0.9800
N1—C7	1.404 (5)	C2—C4	1.529 (6)
O2—C7	1.196 (5)	C2—C6	1.530 (6)
O3—C8	1.194 (5)	C2—H2A	0.9800
O5—C15	1.205 (5)	C14—C13	1.537 (6)
C16—O6	1.203 (5)	C14—H14A	0.9700
C16—N2	1.402 (5)	C14—H14B	0.9700
C16—C12	1.511 (5)	C9—C13	1.526 (6)
C11—C15	1.498 (6)	C9—H9A	0.9800
C11—C9	1.527 (6)	C13—H13A	0.9700
C11—C12	1.536 (5)	C13—H13B	0.9700
C11—H11A	0.9800	C4—H4A	0.9800
N2—C15	1.397 (5)	C6—C5	1.539 (7)
C12—C10	1.533 (6)	C6—H6A	0.9700
C12—H12A	0.9800	C6—H6B	0.9700
C7—C4	1.502 (6)	C5—H5A	0.9700
C1—C3	1.523 (6)	C5—H5B	0.9700
C1—C5	1.525 (6)		
			
C10—O4—C9	96.2 (3)	C1—C3—C4	101.5 (3)
C1—O1—C2	96.2 (3)	C8—C3—H3A	112.3
N2—N1—C8	123.4 (3)	C1—C3—H3A	112.3
N2—N1—C7	121.8 (3)	C4—C3—H3A	112.3
C8—N1—C7	114.6 (3)	O1—C2—C4	101.0 (3)
O6—C16—N2	124.1 (4)	O1—C2—C6	103.4 (4)
O6—C16—C12	129.7 (4)	C4—C2—C6	109.1 (4)
N2—C16—C12	106.1 (3)	O1—C2—H2A	114.0
C15—C11—C9	110.5 (3)	C4—C2—H2A	114.0
C15—C11—C12	105.3 (3)	C6—C2—H2A	114.0
C9—C11—C12	101.0 (3)	C10—C14—C13	101.0 (3)
C15—C11—H11A	113.1	C10—C14—H14A	111.6
C9—C11—H11A	113.1	C13—C14—H14A	111.6
C12—C11—H11A	113.1	C10—C14—H14B	111.6
N1—N2—C15	123.4 (3)	C13—C14—H14B	111.6
N1—N2—C16	120.8 (3)	H14A—C14—H14B	109.4
C15—N2—C16	114.6 (3)	O4—C9—C13	102.3 (3)
C16—C12—C10	110.1 (3)	O4—C9—C11	101.2 (3)
C16—C12—C11	106.3 (3)	C13—C9—C11	111.1 (3)
C10—C12—C11	101.8 (3)	O4—C9—H9A	113.7
C16—C12—H12A	112.6	C13—C9—H9A	113.7
C10—C12—H12A	112.6	C11—C9—H9A	113.7
C11—C12—H12A	112.6	C9—C13—C14	101.9 (3)
O2—C7—N1	123.0 (4)	C9—C13—H13A	111.4
O2—C7—C4	130.2 (4)	C14—C13—H13A	111.4
N1—C7—C4	106.8 (4)	C9—C13—H13B	111.4
O1—C1—C3	102.5 (3)	C14—C13—H13B	111.4
O1—C1—C5	104.4 (4)	H13A—C13—H13B	109.3
C3—C1—C5	107.9 (4)	C7—C4—C2	112.5 (4)
O1—C1—H1A	113.7	C7—C4—C3	105.7 (3)
C3—C1—H1A	113.7	C2—C4—C3	100.9 (3)
C5—C1—H1A	113.7	C7—C4—H4A	112.3
O3—C8—N1	124.5 (4)	C2—C4—H4A	112.3
O3—C8—C3	128.3 (4)	C3—C4—H4A	112.3
N1—C8—C3	107.2 (4)	C2—C6—C5	101.6 (4)
O4—C10—C14	103.6 (3)	C2—C6—H6A	111.4
O4—C10—C12	101.7 (3)	C5—C6—H6A	111.4
C14—C10—C12	109.1 (4)	C2—C6—H6B	111.4
O4—C10—H10A	113.8	C5—C6—H6B	111.4
C14—C10—H10A	113.8	H6A—C6—H6B	109.3
C12—C10—H10A	113.8	C1—C5—C6	101.1 (4)
O5—C15—N2	122.7 (4)	C1—C5—H5A	111.6
O5—C15—C11	129.7 (4)	C6—C5—H5A	111.6
N2—C15—C11	107.5 (3)	C1—C5—H5B	111.6
C8—C3—C1	112.6 (4)	C6—C5—H5B	111.6
C8—C3—C4	105.2 (3)	H5A—C5—H5B	109.4
